# Impact of mining on the floristic association of gold mined sites in Southwest Nigeria

**DOI:** 10.1186/s12898-020-00276-9

**Published:** 2020-02-10

**Authors:** Adegbite A. Adesipo, Sehinde Akinbiola, Olusegun O. Awotoye, Ayobami T. Salami, Dirk Freese

**Affiliations:** 1 Department of Soil Protection and Recultivation, Brandenburg University of Technology Cottbus-Senftenberg, Konrad-Wachsmann-Allee 6, Cottbus Platz der Deutschen Einheit 1, 03046 Cottbus, Germany; 2 Institute of Ecology and Environmental studies, Obafemi Awolowo University, Ile-Ife, Osun State Nigeria; 3 The Technical University (Tech-U), Ibadan, Oyo State Nigeria

**Keywords:** Biodiversity loss, Artisanal and Small-Scale Mining (ASM), Tropics, Functional plants, Ecological indicators, Invasive species

## Abstract

**Background:**

Occurrences in land use, human activities and climate change have both direct and indirect influences on the environment. Of interest for this study is mining; a common activity in developing countries such as Nigeria which is endowed with over 34 solid minerals. The gold mining sites in the Southwest region of the country is predominantly by Artisanal and Small-Scale Mining (ASM). Though the benefits are known, its induced consequences are enormous. To understand its extent of floristic diversity, identification of functional plants and plant species surviving on the mined sites (despite its characterized mining and alteration level); this study compared the floristic composition of an abandoned mining site (Site 1), an active mining site (Site 2) and an undisturbed vegetation sites (Control) of similar vegetation zone.

**Results:**

A total of 54, 28 and 37 species belonging to 31, 20 and 23 families were found on Site 1, Site 2 and the control site, respectively. It shows that the floristic composition of all the sites has been altered due to its past intense agricultural colonization and human activities, but severe on Site 1 and 2 due to mining. Lots of the identified species are functional species and stand as ecological indicators. Species such as *Acanthus montanus* and *Icacina trichantha* found on the Control sites are native and significance but species such as *Capsicum frutescens* and *Crassocephalum crepidioides* on Site 2 are due to human inference while most species on Site 1 shows both original and altered floristic composition (e.g. *Adenia venenata* and *Grewia flavescens*).

**Conclusions:**

Apart from the on-going farming activities, ASM activities such as pollution, deforestation and exposure of the forest soils to direct sunlight has greatly stressed and disturbed the floristic composition, species richness, life form patterns, of the mined sites as well as introduction of non-native plant species. It is therefore necessary to develop effective approaches and policies to curb these illegal ASM activities, empower the community (especially youths), stabilize the economy and establish sustainable development strategies with adequate reclamation measures.

## Background

Biodiversity loss is not just region specific; it is a global challenge and can be attributed to different occurrences in land use, human activities as well as climate changes [14]. Depending on the type and intensity of these occurrences, there are identifiable direct and indirect effects it poses to the environment; they play a crucial role in its dynamics thereby inducing notable quantitative and qualitative changes in the composition of the flora, fauna, biocoenoses, and habitats [21]. Thus, the status of biological diversity in many settlements is a reflection of its environmental conditions, and the susceptibility of each community to the nature and magnitude of these external forces differs [43]. Effects of theses external forces on biological diversity have been studied in varieties of ecosystem such as ecosystem functioning [18], primates [9], coral reefs [16], forest [45, 46] tundra communities [20], and multiple levels such as molecular pathways [42]. The floristic composition is of interest for this study, it is a representative of occurring changes such as the terrain and seasonal variations (e.g. temperature and precipitation). Knowledge about it is essential for many ecological studies such as succession, ecological balance, etc. The natural functions of native species in maintaining ecological balance within an ecosystem remains crucial [12, 47]. The disappearance of these endemic species and the introduction of exotic species alters the ecological balance and composition as well as ecosystem functions and services such as pollination, seed dispersal, decomposition, resilience, disease control, etc. [11]. These categories of plants are known as functional species, their functions cannot be replaced by aliens species, and their existence remain crucial.

In Nigeria, there are several identified factors that are responsible for modifying the original composition (both floristic and structural) of the rainforest. This includes human activities such as agriculture, increase in rural settlements, mining, rural road networks evolution, government policies, etc. [33, 35, 38, 39]. But of importance for this study is mining. Although the benefits of mining are known, its environmental consequences have been of great concern [4, 22, 26]. Examples of its effects include deforestation, exposure of forest soil to direct sunlight, removal of top soil and subsoil, pollution, contamination, and other characterized activities which change the soil physical, chemical and biological properties. However, the level or extent of the impacts in most cases differs, and it depends on the type of mining.

Artisanal and Small-scale Mining (ASM) is the most predominant in the southwest region of Nigeria. It accounts for over 95% of the entire mining activities and has been in existence since 1902 which connotes the advent of the colonial mining [4, 25, 37]. The artisanal miners’ activities are uncoordinated and illegal, majorly practiced by poverty-driven people seeking means of livelihood. Examples of mined minerals within the southwest region include gold, tin, zinc, etc. For the gold mining regions under consideration in this study, spoil heaps (a mixture of overburden soil and rock) with its associated gold waste are deposited on the ground surface after the gold mining, and it is thereafter used for cultivation by the farmers after some abandoned period [37]. These activities, therefore, lead to increase in the gold waste contaminants, especially heavy metals such as Pb, Cu, Cd, Fe, Hg, As, Zn, etc. [32]. In addition to that is the increased solubility of the contaminants; becoming more available for plants uptake and posing great potential of entry into the food chain coupled with its associated effects. The significant impacts of ASM in increasing the environmental problems within this part of the country have been well discussed. The nature of its activities is characterized informal, uncontrollable with great influence on the floristic composition of this area.

Being in the lush tropical rainforest forest estate, this area is known as a biodiversity sanctuary with the complex terrestrial ecosystem [29]. Due to its complex diversity; it is sometimes assumed that there still remains some species yet to be identified. It is part of the agrarian communities typical for growing cash crops such as cocoa and cola-nut, but it has drastically reduced. Most of the cash crop farmlands are gradually becoming normal subsistence farms with the incorporation of food crops such as banana plantation, tuber crops, etc. [6, 37]. Its less productivity can as well be attributed to the presence of contaminants from the gold mining and changes in the soil properties. The typical native species are being endangered; these endemic species are being replaced by exotic/invasive species, and their relics are found existing in patches across the landscape especially in ecologically sensitive environments. Moreover, there are no adequate regulations guiding the conservation of these endangered species within the region. An example is *Milicia excelsa*; listed as an IUCN near threatened species and ought to be worked with based on appropriate permission. Such regulation is necessary, it helps to conserve, regulate and monitor any ecological imbalance. Altering of ecological balance influences the economic, social, environmental and even cultural values of the community. In addition is the level of importance usually attached to some species in providing necessary goods and services; the importance of some of these species to Nigeria was highlighted by Nigeria Fifth National Biodiversity Report. National Reports [28]. These species are also known as functional species as well as ecological indicators. Their loss poses replicable effects of their functions [24, 40]. However, quantifying this ecological imbalance is difficult, but its meaningful characterization is a reflection of its responses to various impacts [39, 41].

This study was therefore conducted in order to understand the impact of mining on the floristic association of the gold mined sites within the southwest region of Nigeria. It aims to know the current status of its floristic diversity, identification of functional plants and ecological indicators as well as characterized plant species surviving on the mined sites (despite its high level of disturbance from the mining activities and contamination). This may also serve as fundamental and applied reasons especially towards reclamation of the mined sites.

## Results

The biological diversity of the 3 studied sites are presented in Table 1. It shows a species richness of 41, 22 and 33 species with recorded total individual ranging from 424, 225 and 336 on Site 1, Site 2 and the reference site, respectively. Permutational multivariate analysis of variance (PERMANOVA) results showed significant differences in species composition between the three sites (Sites—F _2,9_ = 28.953, *p* < 0.001). A pair-wise test further shows significant differences between Site 1 and Site 2 (p = 0.03), between Site 1 and the reference (p = 0.03) and between Site 2 and the reference (p = 0.03). Simple results for species contributions at 50% cut off for low contribution revealed average similarity of 73% for Site 1, 78% for Site 2 and 80% for the reference site. The most contributing species for Site 1 include *Melanthera scandens*, *Melochia corchorifolia*, *Paullinia pinnata*, *Sida pilosa*, *Desmodium adscendens*, all contributing about 23% out of the total 73%. Site 2 were characterised by *Palisota ambigua*, *Justicia insularis*, *Drynaria laurentii*, *Pentodon pentandrus*, *Chromolaena odorata*, *Cissus quadrangularis* and *Sida pilosa* all contributing about 53% of the average similarities within Site 2.

**Table 1:** Biological diversity measurement (alpha and beta diversity), N per site = 4

	Site 1	Site 2	References
1. Community composition			
1A. Species richness (S) ± SE	41.00 ± 1.0	22.00 ± 0.4	33.00 ± 1.2
1B. No. of individuals (N) mean ± SE	106.00 ± 6.7	56.25 ± 1.1	91.50 ± 2.6
2. Diversity			
2A. Pielous evenness (J) ± SE	0.95 ± 0.01	0.95 ± 0.01	0.95 ± 0.01
2B. Shannon (H´loge) ± SE	3.53 ± 0.02	2.93 ± 0.01	3.34 ± 0.3
2C. Simpson (1 − λ) ± SE	0.97 ± 0.01	0.96 ± 0.01	0.97 ± 0.01

**Fig. 1:**
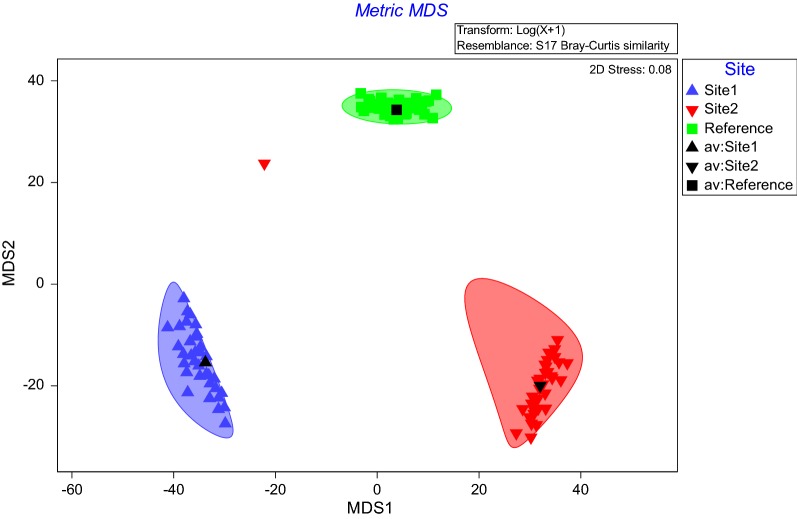
Species composition of the 3 sites by nDMS analysis (with stress value of 0.08). It shows a distinctive separation from the 3 sites: Site 1 (blue); Site 2 (red) and the reference site (green)

**Fig. 2:**
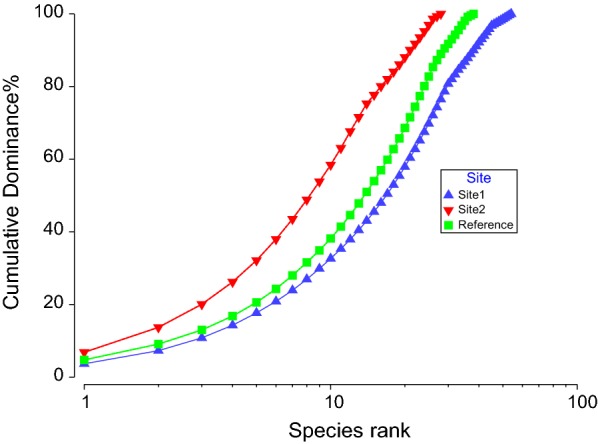
Species dominance curve of the 3 studied sites. The species were ranked on the horizontal axis in terms of abundance and on the vertical axis in terms of percentage dominance with each of the site having different slope

**Fig. 3:**
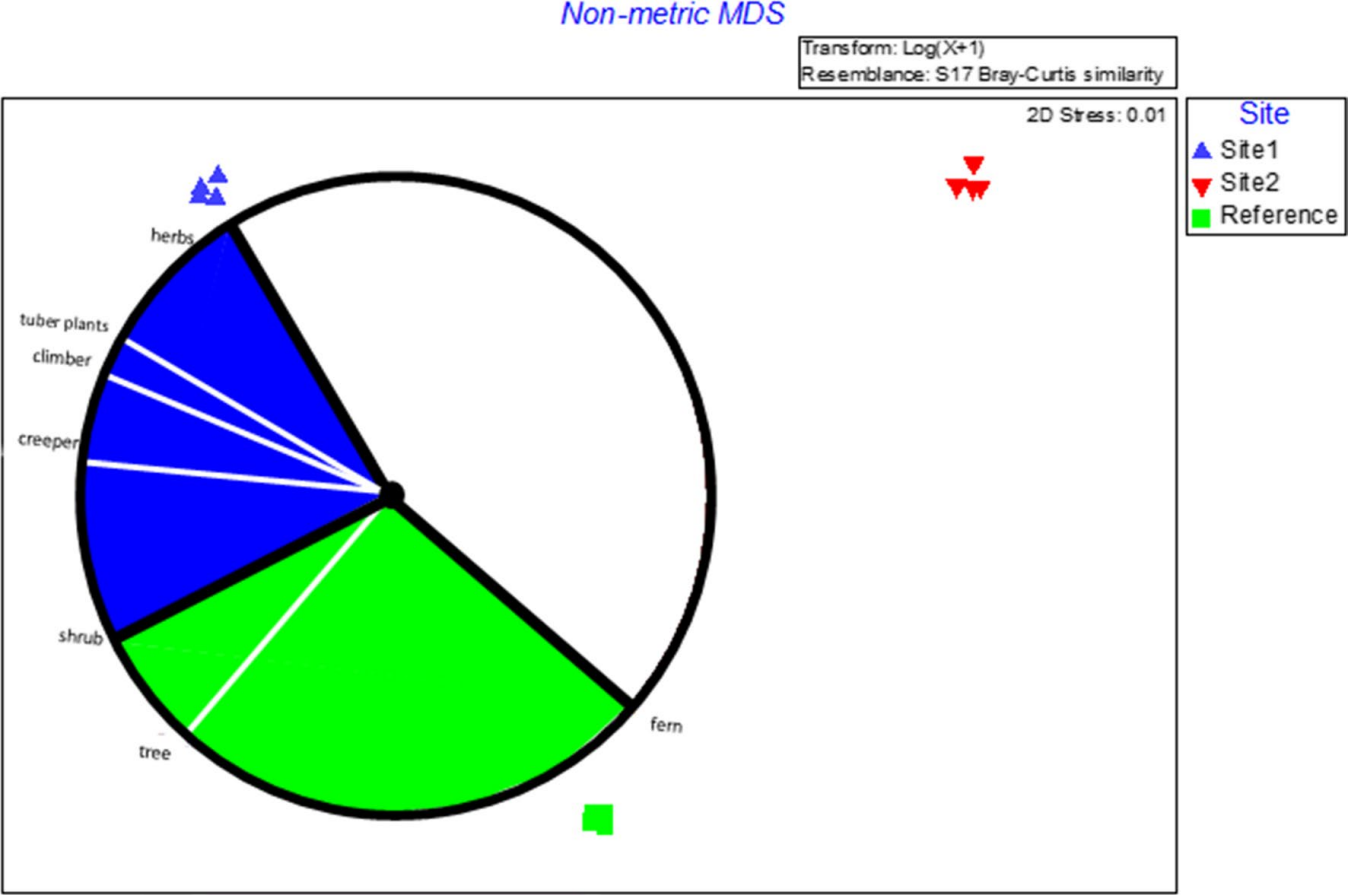
Distinctive association of plant type to each of the 3 studied sites. The reference site (green) is associated with fern, tree and shrub, Site 1 (blue) with herb, tuber plants, climber and creeper while the Site 2 (red) has no distinctive association

The reference site was characterised by *Theobroma cacao*, *Pteris togoensis*, *Crinum jagus*, *Drynaria laurentii*, Justicia insularis all contributing cumulative 23% of the average similarities within the reference site. Site 1 and Site 2 shared about 70% average dissimilarity, while Site 1 and the reference site shared 64% average dissimilarity, and also 64% average dissimilarity exist between Site 2 and the reference site. The Non-metric Multi-Dimensional scaling (nDMS) analysis using bootstrap averages revealed a clear separation of the sites in terms of species composition (Fig. 1) as supported by the stress value (0.08). The three sites are distinctively different with no significant similarity between them. This goes on to confirm the PERMANOVA results that all the three sites in this study are different from each other. Species dominance curve revealed the dominance of most species for all the sites with few rare species (Fig. 2). The species were ranked on the horizontal axis in terms of abundance and on the vertical axis in terms of percentage dominance. And it shows the different slope of the 3 sites. A distinction between the plant types revealed an association of fern, tree and shrub to the reference site (Fig. 3). As presented in Fig. 3, Site 1 was more associated with plant types like herbs, tuber plants, climbers and creepers. No association was however, established for any of the plant types on Site 2. Details of the identified plant species on Site 1, Site 2 and the control site are presented in Tables 2, 3 and 4 respectively. Despite the mentioned (in the methodology) consultations efforts in identifying the plants, 3 plant species were not identifiable on Site 1, while 5 plant species on Site 2 were not identifiable, but all the plant species on the control site were identifiable. The high number of unidentifiable species on the mined sites may be due to its high level of disturbance by the ASM whereby new invasive species might have been introduced to the sites.

**Table 2:** Species and family compositions of flora on Site 1

S/n	Name	Family	Habit	Status
1	*Adenia cissampeloides*	Passifloraceae	Climber	Sparse
2	*Adenia venenata*	Passifloraceae	Climber	Abundant
3	*Albizia leebeck*	Mimosaceae	Tree	Abundant
4	*Albizia zygia*	Mimosaceae	Tree	Abundant
5	*Alchornea cordifolia*	Euphorbiaceae	Shrub	Rare
6	*Alchornea laxiflora*	Euphorbiaceae	Shrub	Abundant
7	*Aneilema aequinoctiale*	Commelianaceae	Climber	Abundant
8	*Aneilema beniniense*	Commelianaceae	Climber	Abundant
9	*Aspilia africana*	Asteraceae	Herb	Abundant
10	*Asystasia gangetica*	Acanthaceae	Herb	Very abundant
11	*Chasmanthera dependens*	Menispermaceae	Climber	Sparse
12	*Chromolaena odorata*	Asteraceae	Herb	Very abundant
13	*Cissampelos owarensis*	Menispermaceae	Climber	Abundant
14	*Cissus quadrangularis*	Vitaceae	Climber	Sparse
15	*Clerodendrum splendens*	Verbenaceae	Climber	Rare
16	*Clerodendrum volubile*	Verbenaceae	Climber	Abundant
17	*Cola nitida*	Sterculiaceae	Tree	Sparse
18	*Colocasia esculentum*	Aracaceae	Herb	Abundant
19	*Commelina erecta*	Commelianaceae	Herb	Abundant
20	*Desmodium adscendens*	Leguminosae	Creeper	Very abundant
21	*Dissotis rotundifolia*	Melastomataceae	Herb	Abundant
22	*Ficus asperifolia*	Moraceae	Shrub	Sparse
23	*Ficus exasperata*	Moraceae	Tree	Sparse
24	*Ficus mucuso*	Moraceae	Tree	Sparse
25	*Gliricidia sepium*	Leguminosae	Shrub	Rare
26	*Grewia flavescens*	Tiliaceae	Shrub	Sparse
27	*Hippocratea indica*	Celastraceae	Climber	Sparse
28	*Holarrhena floribunda*	Apocynaceae	Tree	Sparse
29	*Laportea aestuans*	Urticaceae	Herb	Abundant
30	*Lonchocarpus cyanescens*	Papilionaceae	Tree	Sparse
31	*Manihot esculenta*	Euphorbiaceae	Tuber plant	Abundant
32	*Melanthera scandens*	Asteraceae	Herb	Very abundant
33	*Melochia corchorifolia*	Malvaceae	Herb	Very abundant
34	*Milicia excelsa*	Meliaceae	Tree	Rare
35	*Mondia whitei*	Asclepiadaceae	Climber	Abundant
36	*Morinda lucida*	Rubiaceae	Tree	Sparse
37	*Momordica charantia*	Cucurbitaceae	Climber	Abundant
38	*Mucuna pruriens*	Papilionaceae	Climber	Abundant
39	*Musa paradisiaca*	Musaceae	Shrub	Sparse
40	*Musa sapientum*	Musaceae	Shrub	Abundant
41	*Newbouldia laevis*	Bignoniaceae	Tree	Rare
42	*Paullinia pinnata*	Sapindaceae	Climber	Very abundant
43	*Pouzolzia guineensis*	Urticaceae	Herb	Abundant
44	*Pteris togoensis*	Pteridaceae	Fern	Rare
45	*Senecio biafrae*	Asteraceae	Herb	Sparse
46	*Senna occidentalis*	Caesalpiniaceae	Herb	Rare
47	*Sida acuta*	Malvaceae	Herb	Abundant
48	*Sida pilosa*	Malvaceae	Creeper	Abundant
49	*Spondias mombin*	Anacardiaceae	Tree	Rare
50	*Talinum triangulare*	Portulacaceae	Herb	Abundant
51	*Theobroma cacao*	Sterculiaceae	Tree	Sparse
52	*Triumfetta cordifolia*	Tiliaceae	Herb	Abundant
53	*Vigna unguiculata* (Wild)	Papilionaceae	Climber	Rare
54	*Voacanga africana*	Apocynaceae	Tree	Abundant

**Table 3:** Species and family compositions of flora on Site 2

S/n	Name	Family	Habit	Status
1	*Alchornea cordifolia*	Euphorbiaceae	Tree	Sparse
2	*Acmella oleracea*	Asteraceae	Herb	Sparse
3	*Asystasia gangetica*	Acanthaceae	Herb	Very abundant
4	*Capsicum frutescens*	Solanaceae	Herb	Sparse
5	*Chromolaena odorata*	Asteraceae	Herb	Very abundant
6	*Cissus quadrangularis*	Vitaceae	Climber	Abundant
7	*Clerodendrum volubile*	Verbenaceae	Climber	Sparse
8	*Commelina erecta*	Commelinaceae	Creeper	Abundant
9	*Crassocephalum crepidioides*	Asteraceae	Herb	Sparse
10	*Drynaria laurentii*	Polypodiaceae	Fern	Very abundant
11	*Ficus asperifolia*	Moraceae	Shrub	Sparse
12	*Ficus exasperata*	Moraceae	Tree	Sparse
13	*Justicia insularis*	Acanthaceae	Herb	Very abundant
14	*Laggera pterodonta*	Asteraceae	Herb	Sparse
15	*Melanthera scandens*	Asteraceae	Herb	Abundant
16	*Momordica foetida*	Cucurbitaceae	Climber	Abundant
17	*Musa sapientum*	Musaceae	Herb	Sparse
18	*Palisota ambigua*	Commelinaceae	Herb	Very abundant
19	*Paullinia pinnata*	Sapindaceae	Climber	Abundant
20	*Pentodon pentandrus*	Rubiaceae	Herb	Abundant
21	*Pouzolzia guineensis*	Urticaceae	Herb	Abundant
22	*Pteris togoensis*	Pteridaceae	Fern	Sparse
23	*Rauvolfia vomitoria*	Apocynaceae	Tree	Rare
24	*Sida pilosa*	Malvaceae	Creeper	Abundant
25	*Spilanthes filicaulis*	Asteraceae	Herb	Abundant
26	*Spondias mombin*	Anacardiaceae	Tree	Sparse
27	*Theobroma cacao*	Sterculiaceae	Tree	Sparse
28	*Trema orientalis*	Ulmaceae	Tree	Sparse

**Table 4:** Species and family compositions of flora on control site

S/N	Name	Family	Habit	Status
1	*Acanthus montanus*	Acanthaceae	Herb	Very abundant
2	*Adenia cissampeloides*	Passifloraceae	Climber	Abundant
3	*Adenia lobata*	Passifloraceae	Climber	Abundant
4	*Albizia leebeck*	Mimosaceae	Tree	Abundant
5	*Albizia zygia*	Mimosaceae	Tree	Abundant
6	*Alchornea laxiflora*	Euphorbiaceae	Shrub	Very abundant
7	*Aneilema aequinoctiale*	Commelinaceae	Creeper	Very abundant
8	*Asystasia gangetica*	Acanthaceae	Herb	Abundant
9	*Blepharis maderaspatensis*	Acanthaceae	Creeper	Abundant
10	*Brachiaria deflexa*	Poaceae	Grass	Abundant
11	*Chromolaena odorata*	Asteraceae	Herb	Sparse
12	*Cissampelos owariensis*	Menispermaceae	Climber	Abundant
13	*Cola nitida*	Sterculiaceae	Tree	Abundant
14	*Combretum hispidum*	Combretaceae	Shrub	Sparse
15	*Commelina erecta*	Commelinaceae	Herb	Abundant
16	*Crinum jagus*	Amaryllidaceae	Herb	Very abundant
17	*Drynaria laurentii*	Polypodiaceae	Fern	Very abundant
18	*Elaeis guineensis*	Arecaceae	Tree	Abundant
19	*Ficus exasperata*	Moraceae	Tree	Sparse
20	*Ficus mucoso*	Moraceae	Tree	Sparse
21	*Hewittia sublobata*	Convolvulaceae	Climber	Sparse
22	*Icacina trichantha*	Icacinaceae	Shrub	Sparse
23	*Justicia insularis*	Acanthaceae	Herb	Very abundant
24	*Melanthera scandens*	Asteraceae	Herb	Abundant
25	*Milicia excelsa*	Meliaceae	Tree	Rare
26	*Mondia whitei*	Asclepiadaceae	Climber	Sparse
27	*Musa sapientum*	Musaceae	Herb	Abundant
28	*Paullinia pinnata*	Sapindaceae	Climber	Sparse
29	*Pentodon pentandrus*	Rubiaceae	Herb	Abundant
30	*Phyllanthus amarus*	Euphorbiaceae	Herb	Abundant
31	*Pteris togoensis*	Pteridaceae	Fern	Very abundant
32	*Senecio biafrae*	Asteraceae	Herb	Sparse
33	*Sida acuta*	Malvaceae	Herb	Abundant
34	*Sida pilosa*	Malvaceae	Creeper	Abundant
35	*Sterculia tragacantha*	Malvaceae	Tree	Sparse
36	*Theobroma cacao*	Sterculiaceae	Tree	Very abundant
37	*Voacanga africana*	Apocynaceae	Tree	Abundant

## Discussion

**Fig. 4:**
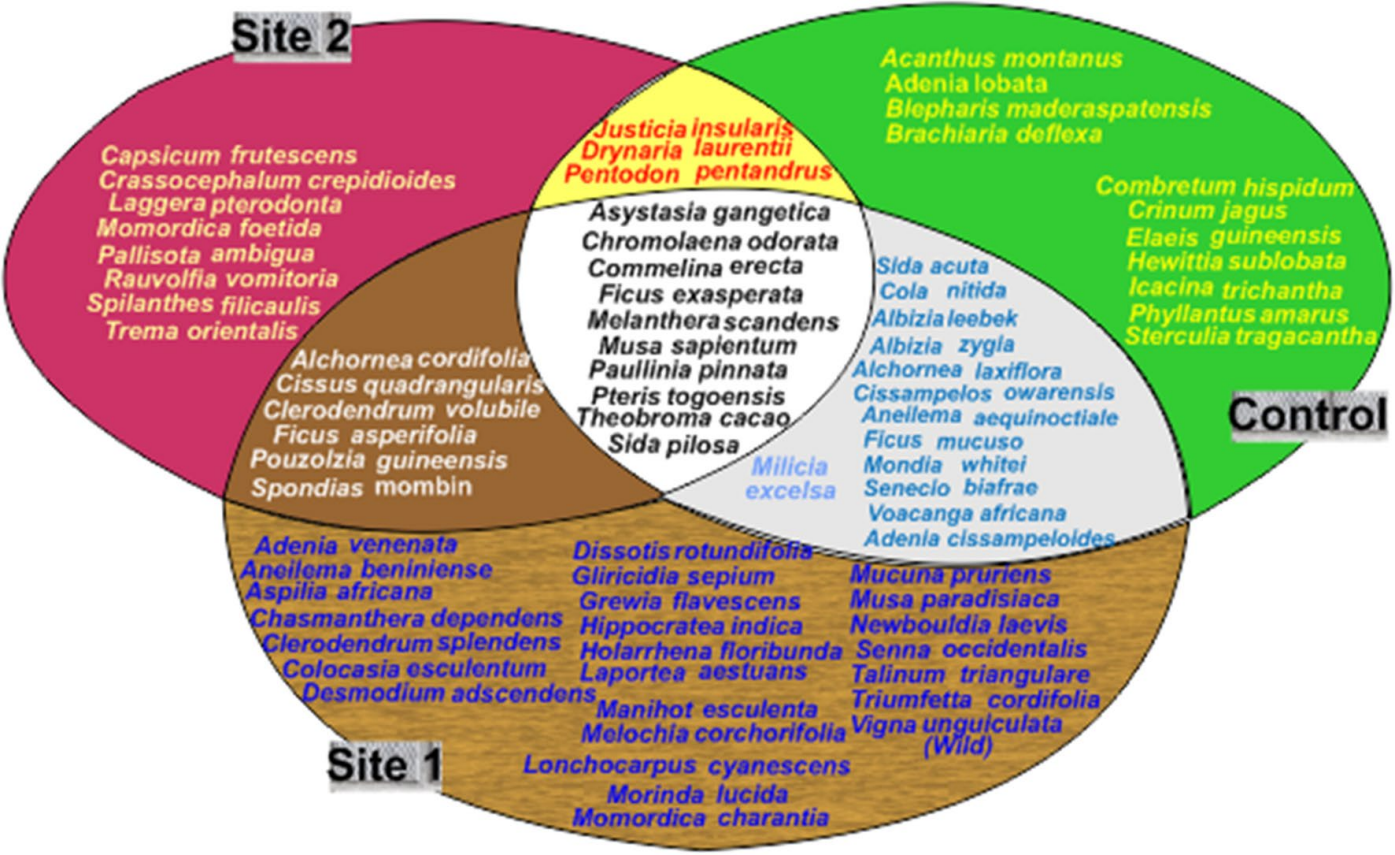
Interrelationship between the Flora species on the 3 sites

For detailed understanding of the interrelationship and species significance of the investigated flora composition for the 3 considered sites; this section discuss the ecological significance of the floristic composition of the plants species identified on the 3 studied sites. Just for clear view, a simple subset diagram was used to present the interrelationships between the sites (Fig. 4).

### All the sites

A total of 10 plant species were common to all the 3 considered sites; they include *Asystasia gangetica*, *Chromolaena odorata*, *Commelina erecta*, *Ficus exasperata*, *Melanthera scandens*, *Musa sapientum*, *Paullinia pinnata*, *Pteris togoensis*, *Sida pilosa* and *Theobroma cacao*. *Musa sapientum* and *Theobroma cacao* are typical plantation crops commonly cultivated in these areas; they are evidence of human activities and intense agricultural colonization that these areas have been subjected to [39]. A*systasia gangetica* is a plantation weed, with wide distribution within this area [17]; they are very abundant on the two mined sites, but just abundant on the control site. Similar to that is *Chromolaena odorata,* it is known as a fast growing perennial shrub, and invasive weeds. It is an aggressive competitor with allelopathy effects. Its sparse status on the control site compared to the two mined sites suggests low level of disturbance [44]. Also widespread in the tropics include *Melanthera scandens*, *Sida pilosa* and *Paullinia pinnata* which is usually in regrowth vegetation, and *Commelina erecta*; particularly in the forest woodland areas. *Ficus exasperata* is a deciduous tree, which belongs to the classified forest communities as noted by Salami [39]. It is found in drier types of forest and also widely distributed [19]. Most of the plant species characterizing the 3 sites are evidences suggesting that the original forest has been subjected to intense agricultural activities which influence the type of floristic association found on the sites.

### Both Site 1 and the Control site

For both Site 1 and the control site; 13 plant species were similar most of which are significant indicators. The species include *Adenia Cissampeloides*, *Albizia leebeck*, *Albizia zygia*, *Alchornea laxiflora*, *Aneilema aequinoctiale*, *Cola nitida*, *Cissampelos owarensis*, *Ficus mucuso*, *Milicia excelsa*, *Mondia whitei*, *Senecio biafrae*, *Sida acuta* and *Voacanga africana. A*ccording to Orwa et al. [34], *Albizia zygia* is a light demanding pioneer species, indigenous to tropical Africa with wide distribution ranging from Senegal to east Africa. It is found in areas of mature secondary regrowth and identified as forest tree capable of ameliorating degraded cocoa farms [8]. Similar to that is *Albizia leebeck*; also naturalized in many tropical countries, found in deciduous and semi-deciduous monsoon forest and rainforest in its native habitat. It is cultivated and often naturalized on old farms. Although these two *Albizia* species are common and abundant on both Site 1 and the control site, however, the species on the control site are fully grown and matured indicating no disturbances unlike Site 1 with young and tender species which are just re-growing due to the mining activities. Likewise, is *Milicia excelsa* (popularly known as Iroko), it grows in deciduous, semi-deciduous or evergreen, primary or secondary forest. It is a considered pioneer species, categorized as Lower Risk/near threatened by the IUCN Red List of Threatened species, with low characterized potential of competition with climbers and shrubs in young secondary forest [48]. Also, typical of this rainforest trees are *Ficus mucuso* and *Cola nitida* [39]. Their absence on Site 2 is suggestive of high level of disturbance. The existence of *Alchornea laxiflora* (an understory shrub) indicates disturbance within the last 100 years [6], while *Voacanga africana* is known to be original occupant of the forest, and species such as *Adenia cissampeloides*, *Sida acuta*, *Cissampelos owarensis*, *Aneilema aequinoctiale* are common and wide spread in the forest regions. *Mondia whitei* is characterized to be rare in rainforest [3, 19, 36].

### Both Site 2 and the Control site

Common to both Site 2 and the control site are only 3 plant species which include *Justicia insularis, Drynaria laurentii* and *Pentodon pentandrus*. All the 3 species are typical for this region with wide distribution. *Justicia insularis* is known as weed of plantation and it is usually characterized with competing potentials [2, 15]. Also is *Drynaria laurentii*; found in the wet tropical environments with native range extending from equatorial Africa to tropical south and East Asia, and *Pentodon pentandrus* found in wet environments, and considered of least concern by IUCN Red List [23].

### Both Site 1 and Site 2

A total of 6 plant species characterized both Sites 1 and 2 where mining activities has been done. They include *Alchornea cordifolia*, *Cissus quadrangularis*, *Clerodendrum volubile*, *Ficus asperifolia*, *Pouzolzia guineensis* and *Spondias mombin.* According to [19], *Spondias mombin* is a deciduous tree, though known as an ancient introduction from America; but also possibly a native of West Africa. It is widespread and common in farmlands, regrowth and villages especially in the forest regions, as well as in the savanna. In fact, it was classified as a savanna species by Salami [39] and areas with its presence were referred to as ecotone community. *Pouzolzia guineensis* is found mainly in the tropics and it is an evidence of succession, while *Ficus asperifolia* is usually found in river areas; its presence on the two mined site is probably because of the availability of water used in washing the mined gold unlike on the control site [19]. *Alchornea cordifolia* is a forest disturbance indicator, largely due to anthropogenic activities, the plant extracts from leaf and back exudates has medicinal properties, hence used locally by villagers. *Clerodendrum volubile* is a climber found in forest or thicket; it is also medicinal and ornamental in nature.

### Only the Control site

On the control site, 11 plant species were observed which include *Acanthus montanus*, *Adenia lobata*, *Blepharis maderaspatensis*, *Brachiaria deflexa*, *Combretum hispidum*, *Crinum jagus*, *Elaeis guineensis*, *Hewittia sublobata*, *Icacina trichantha*, *Pentodon pentandrus*, *Phyllanthus amarus* and *Sterculia tragacantha*. *Acanthus montanus* is majorly found in high forest, with wide distribution within Central and West Africa, it is classified under plant with less concern according to the IUCN, but is easily threatened by land and water pollution Ghogue [13]. This suggests the reason of its inexistence on the two mined sites due to contamination from the gold wastes. Also is *Icacina trichantha*, a small tropical family, in forest and forest regrowth vegetation, found mainly within few countries in West Africa; Nigeria, Ivory Coast, and Benin [19]. *Sterculia tragacantha* is a deciduous tree, remnant of original forest with distributed undergrowth while *Elaeis guineensis* (palm tree), is a cultivated crop (indicating human interference), but its survival on contaminated soils is very low. Also, *Adenia lobata* occurs in the secondary rainforest, while *Combretum hispidum*; a scandent shrub is found majorly in the west part of Nigeria. *Brachiaria deflexa* originated from Africa, and has reached tropics of the new and old world like Middle East, India and south East Asian countries. *Crinum jagus* is a native range of tropical Africa found in the secondary/primary forest, swamp forest, and riverine vegetation while *Phyllanthus amarus*, and *Hewittia sublobata* are widespread in the tropics,

### Only on Site 2

8 different plant species were observed on Site 2 compared to the other two sites. These include *Capsicum frutescens*, *Crassocephalum crepidioides*, *Laggera pterodonta*, *Momordica foetida*, *Pallisota ambigua*, *Rauvolfia vomitoria*, *Spilanthes filicaulis* and *Trema orientalis*. *Capsicum frutescens* is widely dispersed throughout the tropics, cultivated but sometimes naturalized and it is an evidence of human activities [19]. Similar to that is *Crassocephalum crepidioides*, which also indicates very prominent human disturbance. But *Trema orientalis* is an indicator of forest regrowth while *Laggera pterodonta*, *Momordica foetida* are species widespread in tropical Africa and *Pallisota ambigua* in lowland rainforest. *Rauvolfia vomitoria* is also locally called “Asofeyeje” in Yoruba, a shrub common in secondary forest and used medicinally by the locals as medicinal, making of dye, and planted as a shade tree for cocoa.

### Only on Site 1

On Site 1, 25 different species were identified compared to other two sites. These include *Adenia venenata*, *Aneilema beniniense*, *Aspilia africana*, *Chasmanthera dependens*, *Clerodendrum splendens*, *Colocasia esculentum*, *Desmodium adscendens*, *Dissotis rotundifolia*, *Gliricidia sepium*, *Grewia flavescens*, *Hippocratea indica*, *Holarrhena floribunda*, *Laportea aestuans*, *Lonchocarpus cyanescens*, *Manihot esculenta*, *Melochia corchorifolia*, *Morinda lucida*, *Momordica charantia*, *Mucuna pruriens*, *Musa paradisiaca*, *Newbouldia laevis*, *Senna occidentalis*, *Talinum triangulare*, *Triumfetta cordifolia* and *Vigna unguiculata.* Typical plantation crops common in this region which might not have been identified on other sites include *Manihot esculenta* and *Musa paradisiaca*. *Talinum triangulare* (Water leave) is a cosmopolitan/naturalized weed, sometimes cultivated, edible and useful as ethnomedicine [5]. Its presence suggests that this area has been disturbed. But widespread species in the tropics include *Mucuna pruriens*, *Vigna unguiculata* (Wild), *Hippocratea indica. Lonchocarpus cyanescens* (the Yoruba Indigo), *Newbouldia laevis*, and *Clerodendrum splendens*, as well as *Holarrhena floribunda, Morinda lucida, Clerodendrum splendens*, *Aneilema beniniense* in the rainforest, with *Colocasia esculentum* which is native to tropical Asia and South-West Pacific, listed as least concern according to IUCN with no major threats [27]. However, its existence on this site suggests invasive. *Dissotis rotundifolia* are often in damp places as well as *Triumfetta cordifolia*. *Melochia corchorifolia* is common in wet places and widespread in the tropics of the old world, *Cissampelos owariensis* is a twiner mainly in secondary growth in forest regions, while *Adenia venenata* is majorly found in Nigeria; suggesting that this area still maintains some original vegetation significance. But there are some identified savanna plant species which indicate the high intense of disturbance this site has been subjected to. These include *Grewia flavescens*, and *Chasmanthera dependens*, and similar to that are *Momordica charantia*, but *Aspilia africana* is common and widespread in the warmer part of the world.

## Conclusions and recommendations

It could be deduced from the study that the floristic composition of all the 3 sites has been altered with significant differences from one site to the other. This can be attributed to the intense agricultural colonization and human activities on the sites in time past. However, the ASM activities have as well significantly influenced the floristic composition of the mined sites, its species richness, life form patterns, with occurrence of non-native plant species on the mined sites. Many of the differently identified species found on the control sites are native and significance (e.g. *Acanthus montanus* and *Icacina trichantha*), species found on Site 2 (e.g. *Capsicum frutescens* and *Crassocephalum crepidioides*) are due to human inference while the species on Site 1 shows both original and disturbed floristic composition (e.g. *Adenia venenata* and *Grewia flavescens*). In addition is the presence of *Ficus asperifolia* usually associated with gold found on the two mined sites but not on the control site. Though all the studied sites have been stressed; resulting in less number of species characterized for these sites compared to previous studies. However, the mined sites had not only been stressed but had also been greatly disturbed. This can be traced to the on-going farming activities and ASM activities such as pollution, deforestation and exposure of the forest soils to direct sunlight. The stress decreases the biodiversity of the species while the disturbance increases its productivity. The washing and processing of the mined gold might have also influenced the substrate conditions of the mine sites which act as an ‘environmental sieve’ especially for the groundwater. It is therefore necessary to develop effective approaches and policies that will curb illegal ASM activities within the south-west region of Nigeria. In doing this, it is also of importance to investigate its supply chains; this is because several nationalities are sometimes noticed illegally on the sites. The community (especially the youths that are used in the mining activities) need to be empowered to discourage this illegal source of income. Coupled with that is conservation and management measures that can enhance the sustainable development and stabilize the economy of this area, thereby discouraging the continuation of ASM mining and encourage adequate reclamation.

## Methods

### Experimental area

**Fig. 5:**
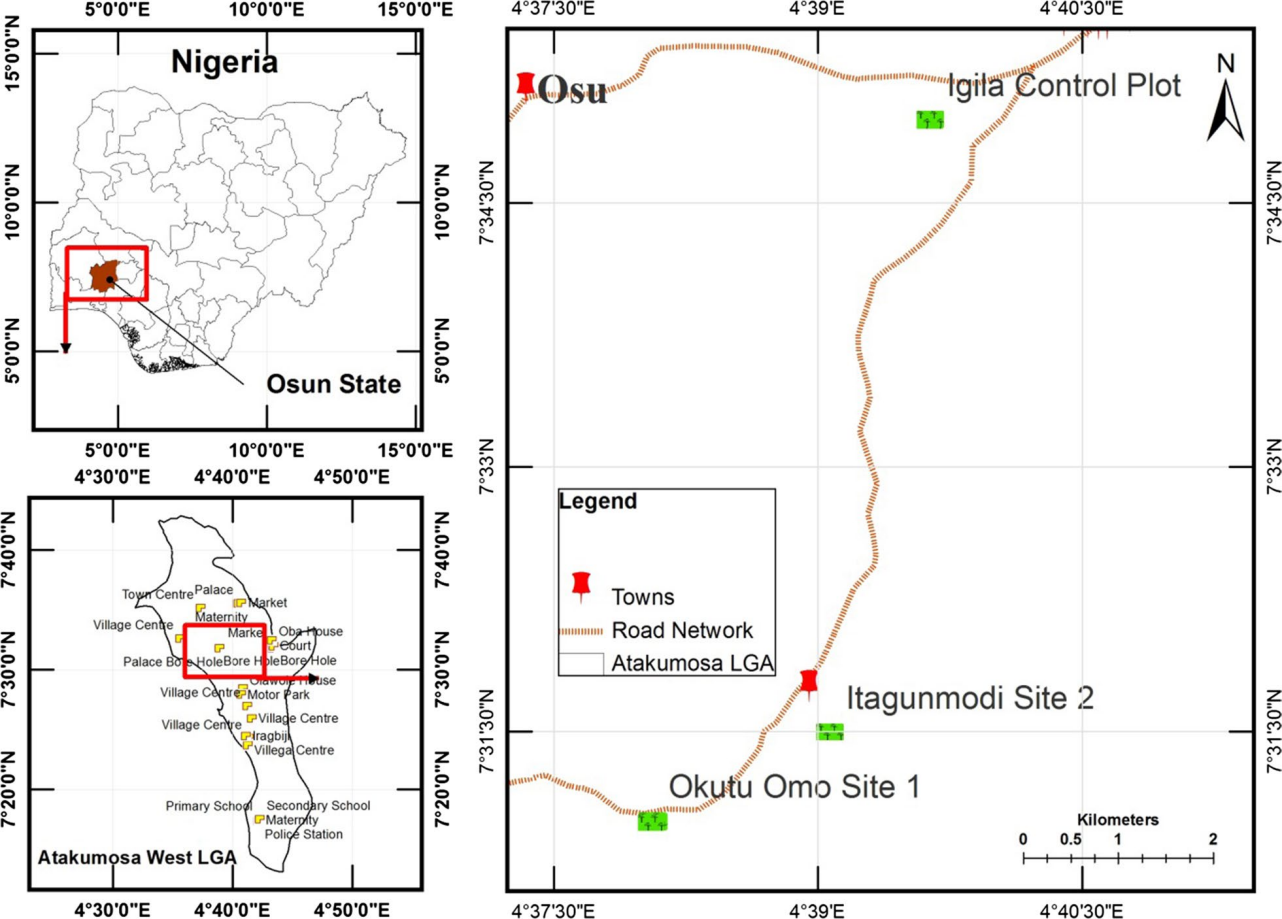
Scaled map of the study areas

The study areas consisted of an abandoned mining site (Site 1) an active mining site (Site 2) and an undisturbed vegetation site for control. Site 1 is situated at Okutu-Omo (7° 30′ 30″ N, 4° 38′ 15″ E), Site 2 at Itagunmodi village (7° 31′ 30″ N, 4° 39′ 03″ E) and the Control site (or reference site) at Igila (7° 34′ 56″ N, 4° 39′ 50″ E) in Atakunmosa west Local Government Area (LGA) of Osun state, within the southwest region of Nigeria (Fig. 5). This area belongs to the Ife-Ilesha schist belts, it remains the main source of both alluvial and primary gold field deposits and the gold mining in this region can be dated back to 1942. The mean annual rainfall is approximately 1400 mm, and the average temperature ranges between 23 and 31 °C. The textural class of the soil series is classified loamy (58% sand, 10% clay and 32% silt), with pH of 4.5 [31], and it is in the lowland rainforest vegetation zone [37]. Despite the characterized mining activities and high level of disturbances, farming remains on-going; since it is the major occupation of the local communities.

### Site selection and preparation

**Fig. 6:**
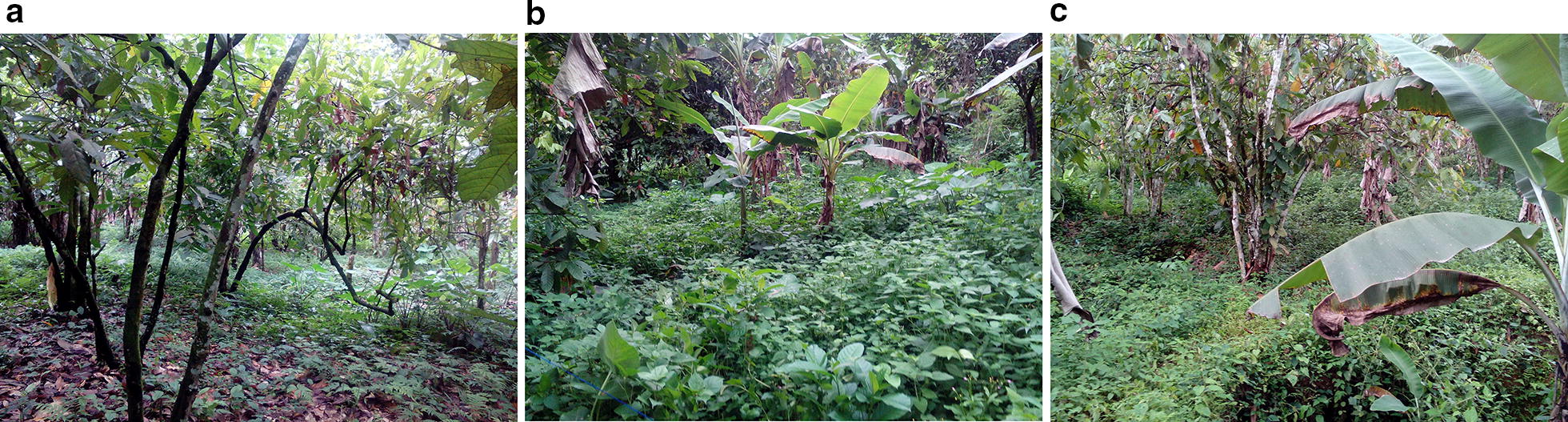
Personally taken pictorial view of the considered sites. **a** Control site **b** Mined site 1 and **c** Mining site 2

Based on reconnaissance surveys, the three sites were purposefully selected to represent the mining and farming activities within this region. As presented in Fig. 6; Site 1 is an old abandoned mined site; occupied by degraded secondary lowland rainforest under regrowth. It had some relics of original forest that were still present. Site 2 is a recent mine site depicted by the presence of pits and spoil heap. It has been subjected to large scale mining and covered with secondary succession plants. Some of the existing pits (about 1.5 × 1 m each) on the site remain uncovered, surrounded by excavated subsoil which are less productive and suggests having influence on plant growth. On the other hand, the control site was an undisturbed vegetation site (also confirmed by the local farmers and inhabitants); it was majorly covered with secondary regrowth forest, located at about 7 km away from the mined sites. Although there were other areas where mining was ongoing, they were not considered due to the suspicious and dangerous disposition of the artisanal miners. Moreover, these 3 considered sites were within the same vegetation zone with similar geology, land use and land cover. On each of the 3 different selected sites, experimental sample plots of 10 × 10 m were mapped out in 2 replicates, and there GPS coordinates were recorded accordingly. Due to the uncontrollable easy access of the ASM miners as well as the local farmers to the sites (especially on Site 2), each of the mapped out sample plots were demarcated from potential invaders throughout the sampling period.

### Plant sampling scheme and classification

**Fig. 7:**
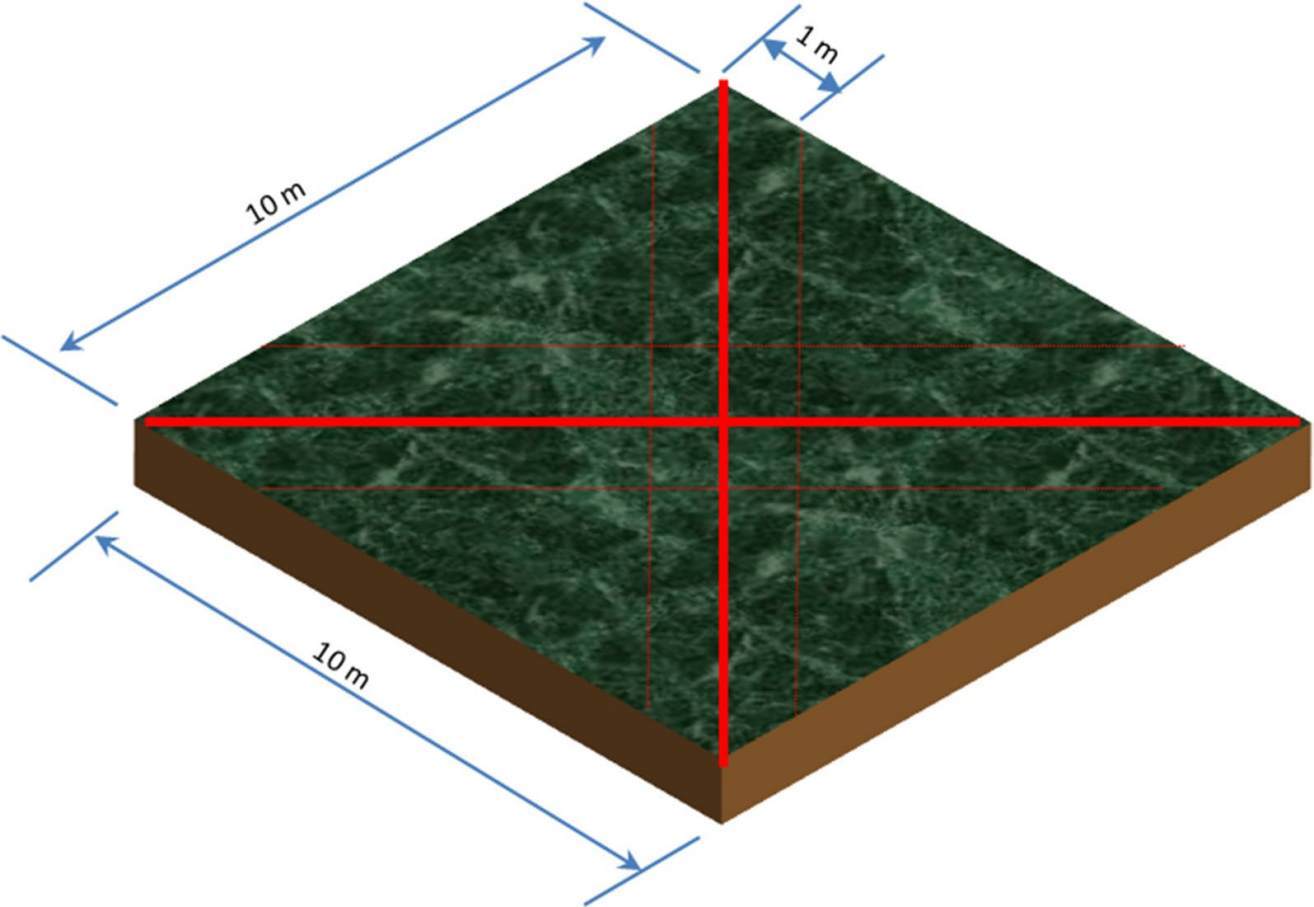
Diagramatic representation of the line transect sampling method

Considering the aim of the study, the plant sampling scheme involves complete enumeration and identification of both lower and higher plants species on each of the mapped out experimental plots. Line transect sampling method was employed; placed on each diagonal edge of the sample plots (Fig. 7) and each line representing a replicates (i.e. total of 4 replicates per site). The line transect was diagonally placed to have a longer sampling length than simple straight line from one edge to the other. All the plant species found at about 1 m on both right and left sides of the lines were recorded. The sampling was done both to and from along the transect line for reaffirmation. Compare to other sampling methods, line transect was employed due to the nature of the landscape and the plant species growing on the sites [10]. This also helps to identify the potential occurrence of changes along the line especially on areas with patchy nature of some plant species. The Herbarium of the Obafemi Awolowo University of Ile-Ife was consulted for unidentified plants coupled with the online portal of The Plant List as well as African Plant Database for confirmation. On each of the sites, all the identified plant species were grouped into their various families and habitat preference. Based on the frequency of the plant species occurrence, they were classified into the different status of abundance ranging from very abundant, abundant, to sparse and rare.

### Data analysis

Plymouth Routines in Multivariate Ecological Research (PRIMER-E) was used for analyzing the data. The significance of difference among the study sites were analyzed using PERMANOVA (Permutational multivariate analysis of variance) and pairwise comparison between the sites. Vegetation data were log transformed (Log(X + 1)) to improve homogeneity of variances. Species richness, number of individuals, Pielous evenness, Shannon and Simpson indices were estimated from the log transformed data with the “DIVERSE” function in PRIMER 7 [7]. A one-factorial Permanova design using Bray–Curtis distance measure and 9999 permutations with site as fixed factor for each site at 0.05 significant level was used to test for the significant differences of plant species composition of each site. Also, a Non-metric Multi-Dimensional scaling (nMDS) based on Bray–Curtis distance measure [30] was created to produce a 2D representation of species composition. Plant types were then overlaid on the nMDS ordination to show linkages between sites and plant types. The goodness of fit of the nDMS results was evaluated with a stress value. The data set employed for the analysis is available in [1].

## Data Availability

The data set used and/or analysed supporting the results of this article are available in the Dryad repository; 10.5061/dryad.xwdbrv19j.

## Abbreviations

ASM: Artisanal and Small-Scale Mining
IUCN: International Union for Conversation of Nature
LGA: Local Government Area
nMDS: Non-metric Multi-Dimensional scaling
PERMANOVA: Permutational multivariate analysis of variance
PRIMER-E: Plymouth Routines in Multivariate Ecological Research
